# The middle-range theory of the nursing diagnosis “ineffective health self-management”

**DOI:** 10.1590/0034-7167-2024-0470

**Published:** 2025-09-08

**Authors:** Rebecca Stefany da Costa, Harlon França de Menezes, Nanete Caroline da Costa Prado, Ana Beatriz Pereira, Thais Targino Ferreira, Jose Rebberty Rodrigo Holanda, Richardson Augusto Rosendo da Silva

**Affiliations:** I Universidade Federal do Rio Grande do Norte. Natal, Rio Grande do Norte, Brazil; II Universidade Federal do Rio Grande do Norte. Calco, Rio Grande do Norte, Brazil

**Keywords:** Nursing Theory, Nursing Diagnosis, HIV, Patient Acceptance of Health Care, Treatment Adherence and Compliance, Teoria de Enfermería, Diagnóstico de Enfermería, VIH, Aceptación de la Atención de Salud, Cumplimiento y Adherencia al Tratamiento

## Abstract

**Objectives:**

to develop a middle-range theory for the nursing diagnosis “ineffective health self-management” in individuals living with the human immunodeficiency virus.

**Methods:**

this is a methodological study aimed at theoretical development to establish the theoretical-causal validity of the nursing diagnosis “ineffective health self-management” based on the theoretical frameworks of Callista Roy and Lopes, Silva, and Herdman. We conducted a scoping review, resulting in a final sample of 31 articles.

**Results:**

the findings identified five essential attributes, 30 antecedents, and nine clinical consequences. We constructed a middle-range theory comprising 1 pictorial diagram, 8 propositions, and 30 causal relationships (15 predisposing factors, 10 disabling factors, 2 precipitating factors, and 3 reinforcing factors).

**Conclusions:**

the development of this theory enables nurses’ clinical judgment regarding the nursing diagnosis “ineffective health self-management” in the context of individuals living with human immunodeficiency virus.

## RESUMEN

**Objetivos:**

desarrollar una teoria de rango medio para el diagnóstico de enfermería “autogestión ineficaz de la salud” en personas que viven con el virus de la inmunodeficiencia humana.

**Métodos:**

estúdio metodológico de desarrollo teórico para la validez teórico-causal dei referido diagnóstico, basado en los marcos de Callista Roy y de Lopes, Silva y Herdman. Se llevó a cabo una revision de alcance con 31 artículos.

**Resultados:**

se identificaron 5 atributos esenciales, 30 antecedentes y 9 consecuencias clínicas. La teoria desarrollada incluye 1 diagrama pictórico, 8 proposiciones y 30 relaciones causales (15 factores predisponentes, 10 incapacitantes, 2 precipitantes y 3 de refuerzo).

**Conclusiones:**

la teoria permite el juicio clínico de los enfermeros sobre el diagnóstico de enfermería “autogestión ineficaz de la salud” en el contexto de las personas que viven con el virus de la inmunodeficiencia humana.

## INTRODUCTION

Although significant progress has been made in the diagnosis, treatment, and prevention of human immunodeficiency virus (HIV), it is estimated that approximately 39 million people have been infected globally by 2023, with 1.3 million new cases and 630,000 deaths attributed to related diseases. These data establish HIV/AIDS as a major global public health issue^(1)^. In Brazil, 489,594 cases of HIV infection were reported between 2007 and June 2023. Between 2020 and 2022, the country experienced a 17.2% increase in cases, with the highest growth in the Northern and Northeastern regions^(2)^.

The main benefit provided by the adoption of this therapy was the extension of life expectancy of individuals diagnosed with this disease, which began to be considered a chronic condition instead of a fatal one^(3)^. However, despite the advances resulting from this therapeutic approach, there are still several obstacles to be overcome. Among them, ineffective self-management of health by the patient during treatment stands out, which imposes challenges on both services and health professionals.

The nursing process (NP) strengthens nursing’s capacity for care planning, promoting humanized, comprehensive, individualized interventions based on scientific knowledge and directed toward specific human responses. In this way, it facilitates the implementation of care that meets the actual needs of people living with HIV (PLHIV)^(4)^. From this perspective, identifying nursing diagnoses (ND) enables implementing interventions that are better suited to human responses and concrete needs. NANDA International (NANDA-I) includes the ND “ineffective health self-management (00276)”, approved in 2020 and revised in 2023 with an evidence level of 3.3, defined as “Unsatisfactory management of symptoms, therapeutic regimen, and lifestyle changes associated with living with a chronic disease”^(5)^.

Thus, revising the ND “ineffective health self-management” becomes necessary, as encouraged by NANDA-I, to strengthen and develop the taxonomy through research conducted by scholars from different countries. Understanding the phenomenon of ineffective health self-management in the context of PLHIV allows nurses to make clinical judgments to develop therapeutic plans tailored to the actual needs of each individual and their environment^(5)^. In turn, through a theoretical framework, nurses can correlate this phenomenon, using it as a tool to interpret, critique, and integrate knowledge. By forming a deductive and interrelated system, theories stimulate research, expand knowledge, and guide studies to describe nursing care and contribute to the development of strengthened knowledge and practiced^(6)^.

Within this context, middle-range theories (MRT) are defined as a set of interrelated ideas focused on a specific phenomenon, aiming to bridge the gap between theory and nursing practice. Furthermore, they contribute to consolidating concepts related to a given nursing phenomenon to support research and professional practiced^(7)^.

Therefore, developing an MRT to validate the ND in this study seeks to construct a theoretical framework that enables the identification of etiology and clinical characteristics associated with the diagnosis. It also aims to establish cause-and-effect relationships that explain the human responses manifested by PLHIV. MRTs serve as an effective strategy to reduce the gap between clinical practice and education by developing key concepts and measurable variables, thereby contributing to personalized critical and clinical reasoning and enhancing the accuracy of the ND^(8, 9, 10, 11, 12)^.

The MRT for the ND “Ineffective Self-Management” of Health for People Living with HIV is a solid, evidence-based tool that assists the NP by identifying the factors that influence the presence of the ND in this specific group, which can influence the maintenance of health and quality of life in the short term, reduce ND-related complications, reduce the number and length of hospitalizations, and reduce the mortality rate^(9)^. In the long term, it impacts health goals and national and global trends in HIV/AIDS^(10)^.

The state of the art presents studies on ineffective self-management in health^(3, 4, 11, 12)^. However, no studies on the development of a middle-range theory focused on the topic were identified in the literature, thus justifying the present study. Given the above, the research question of the study was: How is a middle-range theory configured, focused on the nursing diagnosis ineffective self-management of health for people living with HIV?

## OBJECTIVES

To develop a middle-range theory for the nursing diagnosis “ineffective health self-management” in individuals living with human immunodeficiency virus.

## METHODS

### Ethical aspects

This research complies with Law No. 9,610 of February 19, 1998^(13)^. As a theoretical study, it does not require approval from an ethics committee since it exclusively utilizes secondary and publicly available data without collecting new data directly from individuals. Furthermore, all studies’ authors used in its construction were properly cited, ensuring compliance with copyright regulations.

### Study design and period

We based this methodological study on theoretical-causal validation.To define the development path of a middle-range theory, we followed Roy’s methodological framework^(14)^, as adapted by Lopes, Silva, and Herdman^(8)^. We conducted the study from January to March 2024.

It comprised six consecutive and complementary stages: I) Defining the approach for constructing the middle-range theory; II) Defining key (main) concepts; III) Developing a pictorial diagram; IV) Constructing propositions; V) Establishing causal relationships; and VI) Providing evidence for practice^(8)^.

The first phase began with the development of a scoping review to identify key concepts (essential attributes, antecedents. and consequences), conceptual definitions, and operational definitions, which served as the theoretical framework for the subsequent stages of the middle-range theory’s construction.

We selected The Joanna Briggs Institute (JBI) as the methodological framework for the scoping review, following its six recommended steps: 1) formulation of the guiding question; 2) literature search and sampling; 3) data collection; 4) critical analysis of the included studies; 5) discussion of results; and 6) presentation of the scoping review^(15)^.

The formulation of the guiding question followed the PCC strategy (Population, Concept, and Context) proposed by Levac, Colquhoun, and O’Brien^(16)^, structured as follows: Population – individuals living with HIV; Concept – ineffective health self-management; and Context – all levels of healthcare complexity. Based on this framework, the following research questions were established: What elements influence ineffective health self-management in individuals living with HIV/AIDS? What is the definition of the concept of ineffective health self-management? What are the attributes, antecedents, and consequences of the concept of ineffective health self-management?

We conducted searches in eight different databases (PubMed®, Web of Science, Scopus, CINAHL, Cochrane Library, Scientific Electronic Library Online, and the Latin American and Caribbean Health Sciences Literature) using controlled descriptors combined with Boolean operators, standardized as outlined in the demonstrative framework (Chart 1).

**Chart 1:** Standardization of search descriptors in databases using the PCC strategy

Objective/Problem	Population	Concept	Context
Extraction	*Pessoas vivendo com HIV*	*Autogestão ineficaz da saúde*	*Todos os níveis de complexidade*
Conversion	People living with HIV/Aids	Ineffective self-management of health	All levels of complexity
Combination	Acquired Immunodeficiency Syndrome; HIV; Aids	Patient Acceptance of Health Care; Treatment Adherence and Compliance	Health Services; Primary Health Care
Construction	“Acquired Immunodeficiency Syndrome” OR “HIV” OR “Aids”	“Patient Acceptance of Health Care” OR “Treatment Adherence and Compliance”	“Health Services” OR “Primary Health Carel”
Usage	(“Acquired Immunodeficiency Syndrome” OR “HIV” OR “Aids”) AND (“Patient Acceptance of Health Care” OR “Treatment Adherence and Compliance”) AND (“Health Services” OR “Primary Health Carel”)		

The search and selection process of records was carried out by two Nursing doctoral students, equipped with a specific protocol, created and registered (Open Science Framework identifier 0.17605/OSF.IO/ZEYAD) for this purpose. Impasses were identified and defined through mutual consensus.The selection of studies was initiated by skimming (titles and abstracts) of the records applying the eligibility criteria; the approved records were forwarded to the full reading phase in order to compose the final sample.

**Figure 1:**
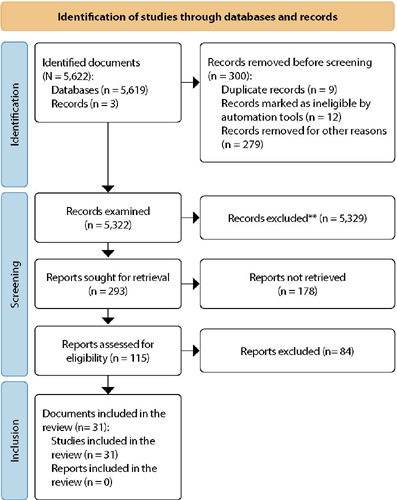
Flowchart of the search and selection process in each database

We determined eligibility criteria based on the research questions. Primary studies addressing ineffective health self-management in patients living with HIV/AIDS, published in Portuguese, English, or Spanish within the past five years, were included. Exclusion criteria encompassed letters to the editor, editorials, review articles, single case studies, abstracts, conference proceedings, books, protocols, undergraduate theses, dissertations, and articles unavailable in full text. We developed the search and sampling process flowchart (Figure 1) in accordance with the Preferred Reporting Items for Systematic Reviews and Meta-Analyses (PRISMA) guidelines.

Data collection was guided by an original protocol, which allowed data tabulation to be established in Microsoft Excel software, version 2013.The following information was extracted from the sample: author(s), year of publication, country of origin of the study, language, journal, scope area of the journal, database, study design, level of evidence, objective of the study, attributes, antecedents, consequences, other concepts and results of the study.

To establish the levels of evidence for the sample, we chose to useThe Joanna Briggs lnstitute^(17)^ as a methodological framework, classifying the studies into five levels: I) Systematic reviews and meta-analyses of randomized clinical trials; II) Randomized clinical trials; III) Cohort studies and case-control studies; IV) Descriptive observational studies; and, V) Expert opinion, committee reports, and research-based theory.

The concepts identified in the SR were analyzed descriptively using a strategy of generalization of specific inferences, resulting in relational and non-relational statements, with a high level of abstraction.

The results were presented in charts and figures, which outline the essential attributes, antecedents and clinical consequences, as well as the causal relationships between the predisposing, disabling, precipitating and reinforcing factors of the ND Ineffective Health Self-Management in people living with HIV, in order to guide the discussion.

In the third phase, we used concept synthesis to create a pictogram-type diagram, which was essential for clarifying the relationships between the concepts and the four adaptation modes (physiological, self-concept, role function, and interdependence) outlined in Callista Roy’s adaptation theory^(18)^.

Thus, we developed propositions between the key concepts based on the scoping review findings, and we established relationships between the concepts based on scientific evidence derived from the review. Finally, we constructed causal relationships from the antecedents, which can provide evidence for applying and testing the developed theory.

## RESULTS

We organized the results according to the stages of developing the MRTfor the nursing diagnosis “ineffective health self-management” in individuals living with HIV, presented sequentially.The process began with defining the approach for constructing the MRT, followed by defining the main (key) concepts, developing a pictorial diagram, formulating propositions, and finally, establishing causal relationships and evidence for practice.

The first phase was assigned to the definition of the TMA construction approach built from the synthesized RE of 31 articles. Characterizing the sample, we have the predominance of articles published in 2020 (18 - 56.2%) and 2021 (10 - 31.25%), cross-sectional design (18 - 56.2%), level of evidence 4 (22 - 68.7%) and 3 (3 - 9.3%), mostly from American countries (14 - 43.7%) and African countries (10 - 31.2%), published in English (22 - 68.7%).

Through the scoping review, we identified five essential attributes based on the fundamental characteristics exhibited by patients diagnosed with ineffective health self-management in the selected studies: multifactorial and dynamic process determining ineffective health self-management (5; 15.6%)^(19, 20, 21, 22, 23)^; abandonment of the therapeutic plan (5; 15.6%)^(23, 24, 25, 26, 27)^; failure to follow recommendations (11; 34.3%)^(20, 24, 28, 29, 30, 31, 32, 33, 34, 35, 36)^; difficulty adhering to received guidance (13; 40.6%)^(19, 22, 37, 38, 39, 40, 41, 42, 43, 44, 45, 46, 47)^; and refusal to follow the therapeutic plan (3; 9.3%)^(21, 48, 49)^. The clustering of these attributes enabled the diagnostic definition of “ineffective health self-management” as “Difficulty, failure, abandonment, or refusal to achieve agreed-upon health goals due to the multifactorial and dynamic process determining ineffective health self-management”.

In addition to the essential attributes, we identified 30 antecedents and 9 clinical consequences, as outlined in Chart 2.

**Chart 2:** Antecedents and clinical consequences included in the middle-range theory for the nursing diagnosis “ineffective health self-management” in individuals living with HIV

Nº	Antecedents	n(%)
01	Alcohol use^(29, 42, 48)^	3 (9.6%)
02	Side effects of ART^(21, 28, 29, 33, 37, 38, 47, 48, 49)^	9 (29%)
03	Illicit drug use^(22, 24, 26, 29, 38, 39, 43, 44, 48, 49)^	10 (32.2%)
04	Stigma and social isolation^(19, 23, 25, 28, 29, 31, 32, 34, 35, 40, 41, 47)^	12 (38.7%)
05	Low education level^(20, 22, 24, 30, 31, 32, 40, 42, 44, 49)^	10 (32.2%)
06	Mental disorders^(20, 22, 26, 28, 31, 32, 36, 37, 38, 41, 42, 43, 47)^	14 (45.1%)
07	Low i ncome^(20, 30, 31, 32, 34, 36, 39, 40, 42, 43, 44, 48)^	12 (38.7%)
08	Barriers to accessing healthcare services^(19, 22, 23, 25, 26, 27, 32, 36, 38, 40, 42, 43, 44, 45, 46, 49)^	16 (51.6%)
09	Lack of support network^(19, 21, 22, 25, 31, 32, 35, 37, 38, 40, 41, 42, 44, 48, 49)^	15 (48.3%)
10	Forgetfulness^(21, 29, 35, 37, 38, 43, 44, 48)^	8 (25.8%)
11	Fear of diagnosis disclosure^(19, 29, 31, 34, 35, 41, 49)^	7 (22.5%)
12	High medication burden^(36, 39, 43, 49)^	4 (12.9%)
13	Race (Black or Brown)^(21, 39, 42)^	3 (9.6%)
14	Denial of disease^(21, 25, 41)^	3 (9.6%)
15	Age < 40 years^(20, 28, 36, 39, 42, 46, 48)^	7 (22.5%)
16	Occupational activity^(24, 29, 30, 31, 40, 41, 46, 49)^	8 (25.8%)
17	Quality of care and/or relationship with healthcare professionals^(21, 23, 25, 28, 30, 31, 32, 38, 40)^	9 (29%)
18	Treatment duration < 1 year^(35, 38, 39, 49)^	4 (12.9%)
19	Treatment duration < 2 years^(19, 37, 40, 48)^	4 (12.9%)
20	Self-centeredness^(21, 48)^	2 (6.4%)
21	Unhealthy environment^(29, 41)^	2 (6.4%)
22	Absence/deficiency of public policies^(24, 27, 30)^	3 (9.6%)
23	Malesex^(20, 21, 26, 32, 39)^	5 (16.2%)
24	Change in therapeutic regimen^(33, 39)^	2 (6.4%)
25	Medication unavailability^(24, 27, 30, 41)^	4 (12.9%)
26	Destabilizing situations^(19, 25, 29, 31, 37, 42, 45)^	7 (22.5%)
27	Food insecurity^(19, 27, 34, 40, 45, 46)^	6 (19.3%)
28	HIV clinical stage (WHO)^(21, 24, 26, 35, 36, 38, 49)^	7 (22.5%)
29	Coinfection (hepatitis and tuberculosis)^(24, 26, 35, 49)^	4 (12.9%)
30	Religion/beliefs opposing the therapeutic plan^(21, 35, 44)^	3 (9.6%)
**Nº**	**Consequences**	**N(%)**
01	Morbimortality^(21, 23, 26, 27, 31, 32, 36, 37, 38, 42, 45, 49)^	13 (41.9%)
02	Disease progression^(21, 23, 26, 27, 31, 32, 36, 37, 38, 42, 45, 49)^	15 (48.3%)
03	Increased susceptibility to opportunistic infections^(28, 30, 34, 35, 36, 38, 40, 46, 48)^	9 (29%)
04	Increased transmissibility potential^(22, 30, 37, 39, 43, 45, 49)^	7 (22.5%)
05	Medication resistance^(21, 22, 23, 26, 28, 29, 31, 32, 33, 34, 36, 38, 39, 42, 44, 46, 49)^	17 (54.8%)
06	Increased viral load^(21, 22, 23, 26, 28, 29, 31, 32, 33, 34, 35, 36, 37, 39, 43, 44, 45)^	17 (54.8 %)
07	Reduced quality of life^(28, 31, 34, 36, 39, 46)^	6 (19.3%)
08	Neurological and/or motor dysfunctions^(6, 20, 22, 31, 42, 48)^	4 (12.9%)
09	Recurrent and/or prolonged hospitalizations^(27, 44)^	2 (6.4%)

**Figure 2:**
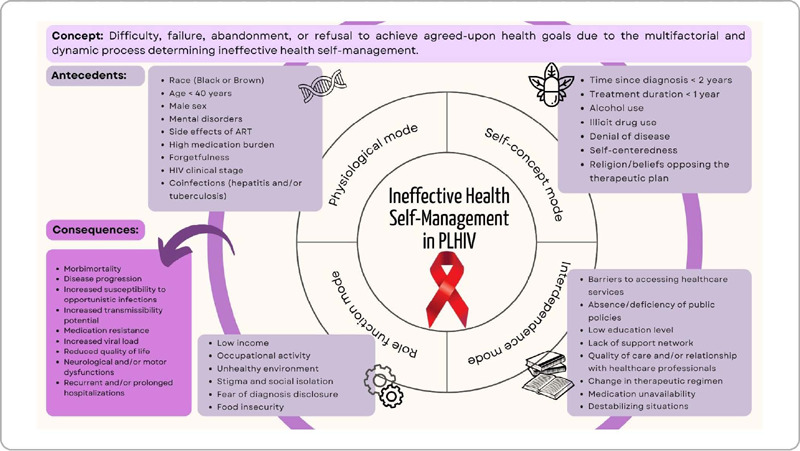
Model pictogram of the middle-range theory for the nursing diagnosis “ineffective health self-management” in individuals living with HIV based on the adaptive modes described in Callista Roy’s adaptation theory

Figure 2 illustrates the relationships established between the antecedents and consequences of the nursing diagnosis “ineffective health self-management” in individuals living with HIV within the perspectives of adaptive coping outlined in Callista Roy’s adaptation theory^(19)^.The figure details how the antecedents are distributed across the four adaptive modes described in the theory: self-concept mode, interdependence mode, role function mode, and physiological mode. Finally, the figure highlights the consequences resulting from this coping process.

To elucidate the causal relationships between antecedents and clinical consequences, eight propositions were formulated, grounded in scientific literature to enhance the understanding of the relationships described in the pictogram (Figure 2). The propositions are as follows:

1. Intrinsic factors such as male sex, age under 40 years, race (Black or Brown), and the presence of mental disorders contribute to ineffective health self-management in individuals living with HIV due to reduced service availability, immaturity, and socioeconomic factors (respectively) during the physiological adaptation phase;
2. Extrinsic factors related to antiretroviral therapy (side effects of ART and high medication burden) contribute to ineffective health self-management in individuals living with HIV because of the high likelihood of gastrointestinal disturbances during the physiological adaptation phase;
3. Factors related to time since diagnosis (less than two years) and treatment duration (less than one year), denial of disease, and self-centeredness contribute to ineffective health self-management in individuals living with HIV due to inexperience in managing a chronic condition combined with social stigma in the self-concept adaptation mode;
4. Lifestyle-related factors (alcohol and illicit drug use) contribute to ineffective health self-management in individuals living with HIV due to the diminished individual capacity for self-care caused by the effects of chemical substances in the self-concept mode;
5. Performance-related factors such as social function (low income, occupational activity, and unhealthy environment) and social perception (stigma and fear of diagnosis disclosure) contribute to ineffective health self-management in individuals living with HIV due to the process of social marginalization in the role function mode;
6. Interdependence factors resulting from social interactions (lack of support network) or the management system (barriers to accessing healthcare services, absence/deficiency of public policies, low education level, medication unavailability, and quality of care received) contribute to ineffective health self-management in individuals living with HIV due to the social and systemic exclusion of vulnerable populations;
7. Ineffective health self-management in individuals living with HIV can lead to systemic damage (neurological and/or motor disorders) and the emergence of opportunistic diseases;
8. Aggravated situations due to ineffective health self-management in individuals living with HIV increase the virus transmissibility rate, contribute to medication resistance, and elevate the number of hospital readmissions, increasing the likelihood of prolonged hospital stays and higher morbidity and mortality rates.

Chart 3 presents the predisposing, disabling, precipitating, and reinforcing factors derived from the antecedents of the nursing diagnosis “ineffective health self-management” in individuals living with HIV. The 30 antecedents were grouped as follows: 15 predisposing factors, 10 disabling factors, 2 precipitating factors, and 3 reinforcing factors.

**Chart 3:** Predisposing, disabling, precipitating, and reinforcing factors of the nursing diagnosis “ineffective health self-management” in individuals living with HIV

**Predisposing factor**	
01	Alcohol use
02	Illicit drug use
03	Stigma and social isolation
04	Low education level
05	Mental disorders
06	Low income
07	Barriers to accessing healthcare services
08	Lack of support network
09	Race (Black or Brown)
10	Denial of disease
11	Age < 40 years
12	Time since diagnosis < 2 years
13	Unhealthy environment
14	Male sex
15	Religion/beliefs opposing the therapeutic plan
**Disabling factor**	
01	Side effects of ART
02	Forgetfulness
03	Fear of diagnosis disclosure
04	High medication burden
05	Quality of care and/or relationship with healthcare professionals
06	Self-centeredness
07	Food insecurity
08	HIV clinical stage (WHO)
09	Coinfection (hepatitis and tuberculosis)
10	Medication unavailability
**Precipitating factor**	
01	Destabilizing situations
02	Change in therapeutic regimen
**Reinforcing factor**	
01	Occupational activity
02	Treatment duration < 1 year
03	Absence/deficiency of public policies

## DISCUSSION

The concept “Difficulty, failure, abandonment, or refusal to achieve agreed-upon health goals due to the multifactorial and dynamic process determining ineffective health self-management”, developed based on the essential attributes identified in the scoping review, encompasses the fundamental characteristics that define ineffective health self-management in individuals living with HIV. These characteristics are essential for recognizing the presence of this nursing diagnosis in this population, highlighting its basic properties (common across all instances) and distinctive features (invariable), which are necessary for subsequent theoretical formulation.

The identification of essential attributes was fundamental for the formulation of the Middle RangeTheory (MRT) for the nursing diagnosis “ineffective health self-management” in individuals living with HIV because it provides a solid and scientific basis for understanding the factors that influence the process of adaptation to therapeutic measures inherent to chronic disease. According to Roy^(18)^, the human capacity for continuous response to internal and external stimuli allows nursing to provide assistance in the adaptation process.

Implementing the concept of “ineffective health self-management” in practice, aligned with the essential attributes and Callista Roy’s adaptation theory^(18)^, requires a holistic approach that includes individualized assessment of patient needs^(19)^, provision of health education^(27)^, development of personalized care plans^(19)^, establishment of continuous support^(33, 49)^, availability of resources and tools^(47)^, and family involvement in the care process. A holistic approach has been shown to yield significant results in self-management among people living with HIV (PLHIV)^(20, 23, 27)^.

In addition to the concept, the identification of patterns and causes of “ineffective health self-management” in individuals living with HIV allows us to understand the variations in the clinical and social context of patients, which enables nurses to personalize care for this specific population, the main characteristic of TMAs. Thus, it was possible to evaluate such patterns and causes by mapping the antecedents of the ND by RE, and then grouping them by adaptive mode: physiological, self-concept, role function and interdependence^(18)^.

The adaptive modes described in adaptation theory^(18)^ represent the different ways in which individuals respond to and adapt to environmental stimuli.The physiological mode addresses basic physical needs. The self-concept mode refers to an individual’s perception and feelings about themselves. The role function mode encompasses an individual’s social roles in life, while the interdependence mode involves social relationships and the support an individual receives and provides.

Grouped in the physiological mode, are the antecedents: black or brown race; age under 40 years; male sex; presence of mental disorders; side effects of antiretroviral therapy (ART); high quantity of medications; pharmacological presentation of medications and forgetfulness regarding the use of ART.

An age of less than 40 years was associated with the nursing diagnosis “ineffective health self-management” in individuals living with HIV due to significantly lower maturity and experience in making health-related decisions for chronic conditions, as cited by several authors^(20, 28, 36, 39, 42, 46, 48)^. Researchers^(20, 21, 26, 32, 39)^ frequently identify male sex as a predisposing factor, several reasons contribute to lower adherence to ART among men^(26)^. The limited number of health services tailored for men and psychosocial factors such as masculinity, cultural aspects, and stigma contribute to treatment dropout and loss to follow-up in this population.

The presence of mental disorders was also identified as a predisposing factor, with depression^(20, 22, 26, 31, 36, 37, 38, 41, 43, 47)^ and anxiety^(28, 3 1, 32, 38, 42, 47)^ keing the most frequently reported. A Canadian cross-sectional study^(33)^ demonstrated a positive association between depression, anxiety, and HIV/AIDS diagnosis, with an incidence rate two to three times higher than in the general population. Another study explains that depression and anxiety diagnoses may interfere with ineffective health self-management in individuals living with HIV due to the extent to which these disorders impair their ability to care for themselves, including adherence to complex treatments^(22, 31)^.

Furthermore, factors associated with the medication (side effects of ART, high quantity of medications and pharmacological presentation) may discourage adherence and/or therapeutic continuity^(21, 28, 29, 33, 37, 38, 47, 48, 49)^. Frequently correlated side effects of ART involve metabolic, neurological and gastrointestinal disorders (nausea, diarrhea, abdominal pain and vomiting)^(29, 37, 38)^, the latter being strongly affected by the presentation of the medications (odor, size and taste)^(38)^ and the high quantity of medications related to the fear of drug-drug interactions^(33, 49)^, essentially in the presence of comorbidities^(28)^. In turn, forgetfulness was associated with the patient’s daily overload (work, study, family)^(21, 29)^, non-use of mechanisms to reinforce the dosage^(35, 44, 48)^, and absence of a motivating support network^(38)^.

Regarding the self-concept mode, the following factors were identified: time since diagnosis less than two years, treatment duration less than one year, alcohol use, illicit substance use, denial of disease, expectation of imminent death, decision-making contrary to the therapeutic plan, false perception of clinical improvement, successive positive laboratory results, religion or beliefs opposing treatment, and secretive medication use.

The greater propensity for the nursing diagnosis “ineffective health self-management” in individuals living with HIV in people with a diagnosis time of less than two years and treatment time of less than one year can be justified by four different factors: the emotional impact of a recent diagnosis^(37, 38, 39, 40)^; the lack of experience in managing a chronic condition^(48, 49)^; the social stigma that makes it difficult to access the necessary support^(19, 35, 48, 49)^; and the complexity of medication regimens and their initial side effects^(37, 40)^ can also be challenging for newly diagnosed patients.

At this point, we highlight the antecedents “alcohol use” and “illicit drug use”, as identified by all authors^(22, 24, 26, 29, 38, 39, 42, 43, 44, 48, 49)^, who described them as the most frequent coping mechanisms following an HIV diagnosis, with significant tendencies toward the nursing diagnosis “ineffective health self-management” in individuals living with HIV. According to the authors^(26, 38)^, these behaviors may serve as a form of “escape” from the previously discussed psychological disorders, which, in turn, diminish an individual’s ability to care for themselves, even when substance use is occasional. Additionally, there is evidence^(44)^ correlating alcohol abuse among PLHIV with the initial phase following HIV diagnosis, when denial of disease^(21, 25, 41)^ and self-centered ness^(21, 48)^ are most frequently observed, both of which have also been identified as antecedents of this nursing diagnosis.

Religion or beliefs contrary to treatment may offer alternative ways of “healing”, and are also related to neglect^(21, 35, 44)^. Neglect of the continued need for adherence to treatment, based only on temporary symptoms or apparent improvements, which leads to complacency and interruption of treatment^(35)^.

Within the interdependence mode, the following antecedents were identified: barriers to accessing healthcare services, absence or inadequacy of public policies, low education level, medication unavailability, diagnostic failures during prenatal care, ineffective supervision by a caregiver, changes in therapeutic regimen, lack of a support network, quality of care and relationships with healthcare professionals, difficulty scheduling appointments and procedures, and social isolation.

The presence of barriers to access to health services may be related to difficulties in obtaining periodic care, viral load monitoring and timely treatment due to socioeconomic, geographic, cultural and travel factors, and the absence or deficiency of public policies that guarantee access to clients^(19, 22, 23, 25, 26, 27, 32, 36, 38, 40, 42, 43, 44, 45, 46, 49)^. Such barriers include other antecedents pointed out in the study in question, such as: unavailability of medications^(24, 27, 30, 41)^ and successive changes in therapeutic regimen by distribution networks^(33, 39)^, difficulty in scheduling appointments and procedures^(43, 48)^, and the quality of care and relationship with health professionals^(25, 26, 27)^. All of these factors were identified as a risk for interruptions and discontinuation of ARTtreatment^(23)^, inadequate self-medication^(25)^, reduced treatment efficacy, and increased risk of developing viral resistance^(44, 49)^.

The antecedent “low level of education” is related to the nursing diagnosis “ineffective health self-management” in individuals living with HIV because it limits the individual’s understanding of the disease, the importance of treatment, and the care needed to effectively manage the condition^(20, 22, 24, 30, 31, 32, 36, 40, 42, 44, 49)^. Low education affects the understanding of medical instructions, the ability to decide on one’s own therapeutic regimen, and access to health services due to linguistic and cultural barriers^(36)^.

Among the factors that reinforce the presence of the antecedent “lack of a support network” and contribute to the presence of “ineffective health self-management” in individuals living with HIV, the most frequently reported were: stimulating emotional support^(19, 21)^, provision of relevant health information^(44)^, assistance with scheduling and monitoring appointments, exams, and medications^(25, 37, 40)^, serving as a potential source of encouragement and motivation^(44)^, and providing access to financial and support resources necessary for ongoing health self-management^(32)^.

Finally, within the role function mode, the following antecedents were identified: low income, occupational activity, unhealthy environment, social stigma and isolation, and fear of diagnosis disclosure. Low income and precarious employment are associated with nursing diagnosis because they limit access to health resources, medication, and professional psychological support^(20, 30, 31, 32, 34, 36, 39, 40, 42, 43, 44, 48)^. Unhealthy environments can aggravate existing health problems and make it difficult to maintain a self-care routine, as is the case for people deprived of liberty^(23, 43)^, transsexuals^(28)^, and sex workers^(29)^. Social stigma has often been associated with nursing diagnosis because it induces isolation and rejection of helphelp^(19, 23, 25, 28, 29, 31, 32, 34, 35, 40, 41, 47)^, while fear of exposing the diagnosis^(19, 29, 31, 34, 35, 41, 49)^ can prevent the search for necessary care^(19, 34)^. These factors interact in a complex way, enhancing a harmful environment and creating substantial barriers to the adaptation of PLHIV to the established therapeutic plan, common to the process of social marginalization.

In the context of nursing practice, as for the physiological mode, interventions should focus on periodic supervision, considering that these factors, although manageable, are diverse and do not directly depend on best healthcare practices despite being monitorable. For the self-concept mode, establishing goals through a dialogical approach fosters trust and integrates practice into the patient’s lived reality, mitigating the challenges associated with a therapeutic regimen that is often inadequate, top-down, and generalized. Regarding the role function mode, multidisciplinaryand interdisciplinary follow-up is essential, based on a comprehensive approach that considers the complexity of the health-disease process and its consequences. Lastly, within the interdependence mode, it is crucial to ensure universal, comprehensive, equitable, and humanized access to healthcare services to prevent social inequalities and the persistence of the Flexnerian model from interfering with health self-management in individuals living with HIV.

Identifying consequential factors is crucial to determine the presence of ND AIS-PLHIV, as these factors provide objective signs and evidence that self-management is compromised^(8, 14)^. They help to understand the specific challenges faced by patients. Understanding these factors allows nurses to perform a more accurate assessment and develop personalized interventions to improve self-management^(3, 4)^. Furthermore, identifying consequential factors can guide health policies and support programs, promoting a more holistic and effective approach to the care of these people, ensuring better adherence to treatment and quality of life^(18)^.

Finally, the pictogram was designed to facilitate the retention of information extracted from this study^(8)^. In some situations, cultural, generational, and educational differences may hinder communication between patients, healthcare professionals, and family members. In this sense, we developed the educational material synthesized in this study to mitigate the risk of communication failures^(8)^ among the key stakeholders in this process, aiming to improve both nursing professionals’ understanding and patient adherence.

With this, the development of the TMA on screen, the nurse will recognize the principles of this phenomenon, since it permeates a variety of demographic and psychosocial factors, where technological care actions can promote effective self-management of treatment^(22, 23, 24)^.The developed construct should have a positive impact on the prevention of comorbidities and complications to which people living with HIV are exposed, thus resulting in an improvement in their quality of life.

### Study limitations

Middle-range theories developed based on scoping reviews often face notable limitations. One of the main challenges is the heterogeneity of sources and data included, which may lead to inconsistencies and difficulties in generalizing findings. Additionally, the varying quality of the studies used in the review can impact the robustness of conclusions, as some research may present methodological biases or inherent limitations. Another challenge is the complexity of synthesizing multiple types of evidence (quantitative and qualitative), which can hinder the development of a cohesive and comprehensive theory.

Finally, reliance on secondary data can limit the depth of analysis because the original data are not directly reassessed, potentially leaving significant gaps in the full understanding of the phenomenon under study. These limitations highlight the need for critical and rigorous approaches when conducting scoping reviews for middle-range theory development.

A specific limitation of this study relates to the exclusive use of open-access articles in the scoping review, which may have resulted in a small sample size. Another limitation was the absence of validation steps necessary to verify the theoretical assumptions against empirical data, preventing potential adjustments and refinements.

### Contributions to the field of nursing

The developed MRT provides a significant contribution to the care of individuals living with HIV, as it supports nurses’ clinical judgment and diagnostic reasoning. Consequently, it assists in planning health promotion actions for this population and in preventing complications related to ineffective health self-management. It is importantto highlightthatthis phenomenon may be associated with nonadherence to antiretroviral therapy, contributing to an increase in new cases of the disease, hospitalizations due to complications, and mortality related to this condition. Furthermore, it advances knowledge of nursing science, by allowing the establishment of a theoretical contribution on the phenomenon studied, proving to be necessary as evidence for updating the NANDA-I taxonomy. Finally, the results can serve as a basis for further studies, in order to advance with content and clinical validation.

## CONCLUSIONS

The development of the middle-range theory allowed for a deeper understanding of the causal relationships between the concepts encompassed in the nursing diagnosis “ineffective health self-management” in individuals living with HIV. Five essential attributes, 35 antecedents, and 19 clinical consequences of the studied nursing diagnosis were identified.To facilitate the understanding of these indicators, an illustrated pictogram was created, depicting the relationship between the concepts (attributes, antecedents, and consequences) and their connection to Callista Roy’s adaptation theory. Finally, seven propositions were developed.

The developed construct should have a positive impact on the prevention of comorbidities and health problems to which people living with HIV are subject and, consequently, on their quality of life. From a broader perspective, it will directly impact the reduction of costs associated with recurrent hospitalizations and high-cost procedures required to treat infections and opportunistic conditions to which individuals with the nursing diagnosis Ineffective Self-Management of Health are predisposed.

Thus, it is concluded that the middle-range theory developed for the nursing diagnosis “ineffective health self-management” in individuals living with HIV will provide valuable contributions to health promotion and the quality of life of this specific population.

## Data Availability

Not applicable.
