# Supplementary material for: The middle-range theory of the nursing diagnosis “ineffective health self-management”
Source: Rev Bras Enferm. 2025 Sep 8;78(3):e20240470. doi: 10.1590/0034-7167-2024-0470 (PMC12419786; doi:10.1590/0034-7167-2024-0470)
Supplement: Translation

# Translation: Teoria de médio alcance do diagnóstico de enfermagem “autogestão ineficaz da saúde”

**DOI:** 10.1590/0034-7167-2024-0470pt

**Authors:** Rebecca Stefany da Costa, Harlon França de Menezes, Nanete Caroline da Costa Prado, Ana Beatriz Pereira, Thais Targino Ferreira, Jose Rebberty Rodrigo Holanda, Richardson Augusto Rosendo da Silva

**Affiliations:** I Universidade Federal do Rio Grande do Norte. Natal, Rio Grande do Norte, Brazil; II Universidade Federal do Rio Grande do Norte. Calco, Rio Grande do Norte, Brazil

**Keywords:** Teoria de Enfermagem, Diagnóstico de Enfermagem, Vírus da Imunodeficiência Humana, Aceitação pelo Paciente de Cuidados de Saúde, Cooperação e Adesão ao Tratamento

## Abstract

**Objetivos:**

desenvolver uma teoria de médio alcance para o diagnóstico de enfermagem “autogestão ineficaz da saúde” em pessoas vivendo com o vírus da imunodeficiência humana.

**Métodos:**

estudo metodológico de desenvolvimento teórico para a validade teórico-causal do diagnóstico de enfermagem “autogestão ineficaz da saúde”, à luz dos referenciais teóricos de Callista Roy e de Lopes, Silva e Herdman. Realizou-se uma revisão de escopo cuja amostra final foi composta por 31 artigos.

**Resultados:**

os resultados convergiram para 5 atributos essenciais, 30 antecedentes e 9 consequentes clínicos. Construiu-se uma teoria de médio alcance formada por: 1 diagrama pictórico ilustrado; 8 proposições; e 30 relações causais (15 fatores predisponentes; 10 fatores incapacitantes; 2 fatores precipitantes; e 3 fatores de reforço).

**Conclusões:**

a criação da teoria possibilita o julgamento clínico dos enfermeiros sobre o diagnóstico de enfermagem “autogestão ineficaz da saúde” no contexto das pessoas vivendo com o vírus da imunodeficiência humana.

## INTRODUÇÃO

Embora tenham sido alcançados avanços significativos no diagnóstico, tratamento e prevenção do vírus da imunodeficiência humana (HIV), estima-se que aproximadamente 39 milhões de pessoas tenham sido infectadas globalmente até 2023, com 1,3 milhão de novos casos e 630 mil mortes atribuídas a doenças relacionadas. Esses dados consolidam o HIV/aids como um problema de saúde pública de grande impacto mundial^(1)^. No Brasil, entre 2007 e junho de 2023, foram registrados 489.594 casos de infecção pelo HIV. Entre 2020 e 2022, o país apresentou um aumento de 17,2% nos casos, com maior crescimento nas Regiões Norte e Nordeste^(2)^.

O principal benefício proporcionado pela adoção dessa terapia foi a extensão da expectativa de vida dos indivíduos diagnosticados com essa enfermidade, que passou a ser considerada uma condição crônica em vez de fatal^(3)^. Contudo, apesar dos avanços decorrentes dessa abordagem terapêutica, ainda existem diversos obstáculos a serem enfrentados. Entre eles, destaca-se a Autogestão Ineficaz da Saúde por parte do paciente durante o tratamento, o que impõe desafios tanto aos serviços quanto aos profissionais de saúde.

O processo de enfermagem (PE) fortalece a capacidade de planejamento assistencial da enfermagem, promovendo intervenções humanizadas, integrais, individualizadas, fundamentadas no conhecimento científico e voltadas para as respostas humanas específicas. Dessa forma, facilita a aplicação de cuidados que atendam às necessidades reais das pessoas vivendo com HIV (PVHIV)^(4)^. Dessa perspectiva, a identificação dos diagnósticos de enfermagem (DE) possibilita a implementação de intervenções mais adequadas às respostas humanas e necessidades concretas. A NANDA Internacional (NANDA-I) aborda o DE “autogestão ineficaz da saúde (00276)”, aprovado em 2020 e revisado em 2023 com nível de evidência 3.3, definido como “Manejo insatisfatório de sintomas, regime terapêutico e mudanças no estilo de vida associados a viver com uma doença crônica”^(5)^.

Dessa forma, torna-se necessária a revisão do DE “autogestão ineficaz da saúde”, encorajada pela NANDA-I visando fortalecer e desenvolver a taxonomia conduzida por pesquisadores de diferentes países. Compreender o fenômeno da autogestão ineficaz da saúde no contexto das pessoas vivendo com HIV permite que o enfermeiro realize um julgamento clínico para desenvolver um plano terapêutico direcionado às reais necessidades de cada indivíduo e do ambiente^(5)^. Por sua vez, mediante uma teoria, o enfermeiro pode correlacionar esse fenômeno, utilizando-a como instrumento para interpretar, criticar e integrar conhecimentos. Ao formar um sistema dedutivo e inter-relacionado, as teorias estimulam a pesquisa, ampliam o conhecimento e orientam o direcionamento dos estudos a fim de descrever o cuidado de enfermagem e contribuir para o desenvolvimento do conhecimento e prática fortalecida^(6)^.

Nesse contexto, encontram-se as teorias de médio alcance (TMA), definidas como um conjunto de ideias relacionadas e focalizadas em um fenômeno, com o objetivo de estreitar a relação entre teoria e prática na enfermagem. Além disso, contribuem para a união dos conceitos sobre determinado fenômeno de enfermagem, para subsidiar a pesquisa e a prática profissional^(7)^.

Portanto, a elaboração de uma TMA para validar o DE no presente estudo visa construir um arcabouço teórico que permita identificar a etiologia e as características clínicas associadas ao diagnóstico. Busca também estabelecer relações de causa e efeito que expliquem as respostas humanas manifestadas por pessoas vivendo com HIV. As TMAs tornam-se estratégia eficaz para reduzir a distância entre a prática clínica e o ensino por meio do desenvolvimento de conceitos-chave e variáveis mensuráveis, buscando contribuir no raciocínio crítico e clínico personalizado e promovendo a precisão do DE^(8, 9, 10, 11, 12)^.

A TMA para o DE Autogestão Ineficaz da Saúde para pessoas vivendo com HIV é uma ferramenta sólida, baseada em evidências, que auxilia o PE através da identificação dos fatores que influenciam para a presença do DE nesse grupo específico, podendo influenciar à curto prazo sobre a manutenção da saúde e qualidade de vida, redução dos agravos relacionados ao DE, redução no número e tempo de internações, e reduzindo a taxa de mortalidade^(9)^. A longo prazo, impacta nas metas em saúde e tendências nacionais e globais sobre a HIV/Aids^(10)^.

O estado da arte apresenta estudos sobre a autogestão ineficaz em saúde^(3, 4, 11, 12)^. Entretanto, não foram identificados na literatura estudos sobre o desenvolvimento de uma teoria de médio alcance voltada ao tema, justificando assim o presente estudo. Diante do exposto, a questão de pesquisa do estudo foi: Como se configura uma teoria de médio alcance, voltada ao diagnóstico de enfermagem autogestão ineficaz da saúde de pessoas vivendo com HIV?

## OBJETIVOS

Desenvolver uma teoria de médio alcance para o diagnóstico de enfermagem “autogestão ineficaz da saúde” em pessoas vivendo com o vírus da imunodeficiência humana.

## MÉTODOS

### Aspectos éticos

A presente pesquisa está em conformidade com a Lei Nº 9.610, de 19 de fevereiro de 1998^(13)^: trata-se de um estudo de natureza teórica que dispensa o aval do Comitê de Ética por utilizar dados secundários e publicamente disponíveis, sem envolver a coleta de novos dados diretamente de indivíduos. Além disso, todos os autores dos estudos utilizados na sua construção foram devidamente citados, respeitando os direitos autorais.

### Desenho e período do estudo

Este estudo metodológico baseou-se na validação teórico-causal. Para definir o percurso de desenvolvimento de uma TMA, seguiu-se o referencial metodológico de Roy^(14)^ adaptado por Lopes, Silva e Herdman^(8)^. O presente estudo transcorreu de janeiro a março de 2024.

Contemplou seis etapas consecutivas e complementares: I) Definição da abordagem para construção da teoria de médio alcance; II) Definição de conceitos-chave (principais); III) Desenvolvimento um diagrama pictórico; IV) Construção de proposições; V) Estabelecimento de relações causais; e VI) Evidências para a prática^(8)^.

A primeira fase foi iniciada pelo desenvolvimento de uma revisão de escopo (RE) a fim de identificar os conceitos-chave (atributos essenciais, antecedentes e consequentes), as definições conceituais e as definições operacionais que serviram de arcabouço teórico para as etapas subsequentes de elaboração da TMA.

The Joanna Briggs Institute (JBI) foi elencado como referencial metodológico para a revisão de escopo conforme as seis etapas recomendadas: 1) elaboração da pergunta norteadora; 2) busca ou amostragem na literatura; 3) coleta dos dados; 4) análise crítica dos estudos que foram incluídos; 5) discussão dos resultados; 6) apresentação da revisão de escopo^(15)^.

A elaboração da questão norteadora seguiu a estratégia PCC (População, Conceito e Contexto) de Levac, Colquhoun e O’Brien^(16)^ com a seguinte disposição: População – pessoas vivendo com HIV; Conceito – autogestão ineficaz da saúde; e Contexto – todos os níveis de complexidade. Desse modo, as seguintes questões de pesquisa foram estabelecidas: Que elementos influenciam a autogestão ineficaz da saúde em pessoas que vivem com HIV/aids? Qual a definição do conceito de autogestão ineficaz da saúde? Quais os atributos, antecedentes e consequentes do conceito de autogestão ineficaz da saúde?

As buscas foram realizadas em oito bases de dados diferentes (PubMed®, *Web of Science,* Scopus, CINAHL, *Cochrane Library, Scientific Electronic Library Online* e Literatura Latino-Americana e do Caribe em Ciências da Saúde) usando os descritores controlados, conjugados por meio dos operadores booleanos e padronizados conforme o quadro demonstrativo (Quadro 1).

**Quadro 1:** Padronização de descritores de busca nas bases de dados usando a estratégia PCC

Objetivo/Problema	População	Conceito	Contexto
Extração	Pessoas vivendo com HIV	Autogestão ineficaz da saúde	Todos os níveis de complexidade
Conversão	*People living with HIV/Aids*	*Ineffective self-management of health*	*All levels of complexity*
Combinação	*Acquired Immunodeficiency Syndrome; HIV; Aids*	*Patient Acceptance of Health Care; Treatment Adherence and Compliance*	*Health Services; Primary Health Care*
Construção	*“Acquired Immunodeficiency Syndrome”* OR “*HIV”* OR “Aids”	*“Patient Acceptance of Health Care”* OR “*Treatment Adherence and Compliance”*	*“Health Services”* OR “*Primary Health CareI”*
Uso	(“*Acquired Immunodeficiency Syndrome” OR “HIV”* OR “Aids”) AND (“*Patient Acceptance of Health Care”* OR “*Treatment Adherence and Compliance”*) AND (“*Health Services”* OR “*Primary Health CareI”*)		

O processo de busca e seleção dos registros foi realizado por dois doutorandos de Enfermagem, munidos de protocolo específico, criado e registrado (*Open Science Framework* identificador 0.17605/OSF.IO/ZEYAD) para este fim. Os impasses foram identificados e definidos através de consenso mútuo. A seleção dos estudos foi iniciada pela leitura flutuante (títulos e resumos) dos registros aplicando os critérios de elegibilidade, os registros aprovados foram encaminhados para a fase de leitura integral, a fim de compor a amostra final.

Os critérios de elegibilidade foram determinados com base nas questões de pesquisa. Foram incluídos estudos primários que abordavam a autogestão ineficaz da saúde em pacientes vivendo com HIV/Aids, publicados em português, inglês ou espanhol, nos últimos cinco anos. A exclusão contemplou cartas ao editor, editoriais, artigos de revisão, estudos de casos únicos, resumos, anais, livros, protocolos, trabalhos de conclusão de curso, teses, dissertações e artigos não disponíveis na íntegra. O fluxograma do processo de busca e amostragem (Figura 1) foi elaborado de acordo com as diretrizes *Preferred Reporting Items for Systematic Reviews and Meta-Analyses* (PRISMA).

**Figura 1:** Fluxograma do processo de busca e seleção em cada base de dados

A coleta de dados foi orientada por protocolo original, os quais permitiu a conformação da tabulação de dados no *software* Microsoft Excel, versão 2013. Foram extraídas da amostra as seguintes informações: autor(es), ano de publicação, país de origem do estudo, idioma, periódico, área de escopo do periódico, base de dados, desenho do estudo, nível de evidência, objetivo do estudo, atributos, antecedentes, consequentes, outros conceitos e resultados do estudo.

Para o estabelecimento dos níveis de evidência da amostra, optou-se por utilizar o *The Joanna Briggs Institute* ^(17)^ como referencial metodológico, classificando os estudos em cinco níveis: I) Revisões sistemáticas e meta-análises de ensaios clínicos randomizados; II) Ensaios clínicos randomizados; III) Estudos de coorte e estudos de caso-controle; IV) Estudos observacionais descritivos; e, V) Opinião de especialistas, relatórios de comitês, e teoria baseada em pesquisa.

Os conceitos identificados na RE foram analisados de forma descritiva por estratégia de generalização de inferências específicas, resultando em afirmativas relacionais e não relacionais, com nível de abstração elevado.

Os resultados foram apresentados em quadros e figuras, os quais delineiam os atributos essenciais, antecedentes e consequentes clínicos, bem como as relações causais entre os fatores predisponentes, incapacitantes, precipitantes e de reforço do DE Autogestão Ineficaz da Saúde em pessoas vivendo com o HIV, a fim de nortear a discussão.

Na terceira fase, a síntese dos conceitos foi utilizada para criar um diagrama do tipo pictograma ilustrado, relevante para clarificar as relações existentes entre os conceitos e os quatro modos de adaptação (necessidades fisiológicas, autoconceito, função de papel e interdependência) constantes na teoria da adaptação, de Callista Roy^(18)^.

Assim, foram criadas as proposições entre os conceitos-chave com base nos achados da RE e foram estabelecidas as relações entre os conceitos com base nas evidências científicas oriundas da revisão. Por fim, foram elaboradas as relações causais, originadas dos antecedentes, o que pode fornecer evidências para praticar e testar a teoria construída.

## RESULTADOS

Os resultados foram organizados conforme as etapas do desenvolvimento da TMA para o DE “autogestão ineficaz da saúde” em pessoas vivendo com HIV, sendo apresentados de forma sequencial. Iniciou-se com a definição da abordagem para a construção da TMA, seguida pela definição dos conceitos principais (conceitos-chave), construção de um esquema pictórico, elaboração das proposições e, finalmente, estabelecimento das relações causais e evidência para a prática.

A primeira fase atribuída à definição da abordagem de construção da TMA construída a partir da RE sintetizada de 31 artigos. Caracterizando a amostra temos o predomínio de artigos publicados em 2020 (18 – 56,2%) e 2021 (10 – 31,25%), de desenho transversal (18 – 56,2%), nível de evidência 4 (22 – 68,7%) e 3 (3 – 9,3%), em sua maioria oriundos de países americanos (14 – 43,7%) e africanos (10 – 31,2%), publicados em língua inglesa (22 – 68,7%).

Com a RE, foram identificados cinco atributos essenciais com base nas características fundamentais apresentadas pelos pacientes diagnosticados com autogestão ineficaz da saúde nos textos da amostra da RE: Processo multifatorial e dinâmico que determina a autogestão ineficaz da saúde (5; 15,6%)^(19, 20, 21, 22, 23)^ Abandono do plano terapêutico (5; 15,6%)^(23, 24, 25, 26, 27)^; Falha em seguir as recomendações (11; 34,3%)^(20, 24, 28, 29, 30, 31, 32, 33, 34, 35, 36)^; Dificuldade em seguir as orientações recebidas (13; 40,6%)^(19, 22, 37, 38, 39, 40, 41, 42, 43, 44, 45, 46, 47)^; e Recusa em seguir o plano terapêutico (3; 9,3%)^(21, 48, 49)^. O agrupamento desses atributos tornou possível a definição diagnostica de “autogestão ineficaz da saúde” como “Dificuldade, falha, abandono ou recusa em alcançar as metas pactuadas devido ao processo multifatorial e dinâmico que determina a autogestão ineficaz da saúde”.

Além dos atributos essenciais, foram mapeados 30 antecedentes e 9 consequentes clínicos dispostos no Quadro 2.

**Quadro 2:** Antecedentes e consequentes clínicos incluídos na teoria de médio alcance do diagnóstico “autogestão ineficaz da saúde” em pessoas vivendo com HIV

Nº	Antecedentes	n(%)
01	Uso de álcool^(29, 42, 48)^	3 (9,6%)
02	Efeitos colaterais da TARV^(21, 28, 29, 33, 37, 38, 47, 48, 49)^	9 (29%)
03	Uso de drogas ilícitas^(22, 24, 26, 29, 38, 39, 43, 44, 48, 49)^	10 (32,2%)
04	Estigma e isolamento social^(19, 23, 25, 28, 29, 31, 32, 34, 35, 40, 41, 47)^	12 (38,7%)
05	Baixo nível de escolaridade^(20, 22, 24, 30, 31, 32, 40, 42, 44, 49)^	10 (32,2%)
06	Transtornos mentais^(20, 22, 26, 28, 31, 32, 36, 37, 38, 41, 42, 43, 47)^	14 (45,1%)
07	BaiXa renda^(20, 30, 31, 32, 34, 36, 39, 40, 42, 43, 44, 48)^	12 (38,7%)
08	Barreiras de acesso aos serviços de saúde^(19, 22, 23, 25, 26, 27, 32, 36, 38, 40, 42, 43, 44, 45, 46, 49)^	16 (51,6%)
09	Ausência de rede de apoio^(19, 21, 22, 25, 31, 32, 35, 37, 38, 40, 41, 42, 44, 48, 49)^	15 (48,3%)
10	Esquecimento^(21, 29, 35, 37, 38, 43, 44, 48)^	8 (25,8%)
11	Medo de exposição do diagnóstico^(19, 29, 31, 34, 35, 41, 49)^	7 (22,5%)
12	Quantidade elevada de medicamentos^(36, 39, 43, 49)^	4 (12,9%)
13	Raça (preta ou parda)^(21, 39, 42)^	3 (9,6%)
14	Negação da doença^(21, 25, 41)^	3 (9,6%)
15	Idade < 40 anos^(20, 28, 36, 39, 42, 46, 48)^	7 (22,5%)
16	Atividade ocupacional^(24, 29, 30, 31, 40, 41, 46, 49)^	8 (25,8%)
17	Qualidade do cuidado e/ou relação com os profissionais de saúde^(21, 23, 25, 28, 30, 31, 32, 38, 40)^	9 (29%)
18	Tempo de tratamento < 1 ano^(35, 38, 39, 49)^	4 (12,9%)
19	Tempo de diagnóstico < 2 anos^(19, 37, 40, 48)^	4 (12,9%)
20	*Si-mismo* ^(21, 48)^	2 (6,4%)
21	Ambiente insalubre^(29, 41)^	2 (6,4%)
22	Ausência/deficiência de políticas públicas^(24, 27, 30)^	3 (9,6%)
23	Sexo masculino^(20, 21, 26, 32, 39)^	5 (16,2%)
24	Troca de regime terapêutico^(33, 39)^	2 (6,4%)
25	Indisponibilidade de medicamentos^(24, 27, 30, 41)^	4 (12,9%)
26	Situações desestabilizadoras^(19, 25, 29, 31, 37, 42, 45)^	7 (22,5%)
27	Insegurança alimentar^(19, 27, 34, 40, 45, 46)^	6 (19,3%)
28	Estágio clínico do HIV (OMS)^(21, 24, 26, 35, 36, 38, 49)^	7 (22,5%)
29	Coinfecção (hepatite e tuberculose)^(24, 26, 35, 49)^	4 (12,9%)
30	Religião/crença contrária ao plano terapêutico^(21, 35, 44)^	3 (9,6%)
Nº	Consequentes	N(%)
01	Morbimortalidade^(21, 23, 26, 27, 31, 32, 36, 37, 38, 42, 45, 49)^	13 (41,9%)
02	Progressão da doença^(21, 23, 26, 27, 31, 32, 36, 37, 38, 42, 45, 49)^	15 (48,3%)
03	Maior susceptibilidade a infecções oportunistas^(28, 30, 34, 35, 36, 38, 40, 46, 48)^	9 (29%)
04	Aumento do potencial de transmissibilidade^(22, 30, 37, 39, 43, 45, 49)^	7 (22,5%)
05	Resistência à medicação^(21, 22, 23, 26, 28, 29, 31, 32, 33, 34, 36, 38, 39, 42, 44, 46, 49)^	17 (54,8%)
06	Aumento da carga viral^(21, 22, 23, 26, 28, 29, 31, 32, 33, 34, 35, 36, 37, 39, 43, 44, 45)^	17 (54,8 %)
07	Redução da qualidade de vida^(28, 31, 34, 36, 39, 46)^	6 (19,3%)
08	Disfunções neurológica e/ou motora^(6, 20, 22, 31, 42, 48)^	4 (12,9%)
09	Internações recidivas e/ou prolongadas^(27, 44)^	2 (6,4%)

A Figura 2 ilustra as relações estabelecidas entre os antecedentes e consequentes do DE “autogestão ineficaz da saúde” em pessoas vivendo com HIV dentro das perspectivas de enfrentamento adaptativo presentes na teoria da adaptação, de Callista Roy^(19)^. A figura detalha como os antecedentes se distribuem nos quatro modos adaptativos descritos pela teoria, sendo estes: modo de autoconceito, modo de interdependência, modo de desempenho e modo fisiológico. Por fim, a figura destaca os consequentes decorrentes desse processo de enfrentamento

**Figura 2:** Pictograma-modelo da teoria de médio alcance do diagnóstico de enfermagem “autogestão ineficaz da saúde” em pessoas vivendo com HIV com base nos modos adaptativos constantes na teoria da adaptação, de Callista Roy

Para elucidar as relações de causalidade entre os antecedentes e consequentes clínicos, foram formuladas oito proposições, as quais foram fundamentadas na literatura científica para proporcionar a compreensão das relações descritas no pictograma (Figura 2). Seguem as proposições:

1. Fatores intrínsecos como sexo masculino, idade inferior a 40 anos, raça (preta ou parda) e a presença de transtornos mentais contribuem para a autogestão ineficaz da saúde em pessoas vivendo com HIV em decorrência da menor oferta de serviço reduzido, imaturidade e fatores socioeconómicos (respectivamente) durante o período adaptativo fisiológico;
2. Fatores extrínsecos relacionados aos antirretrovirais (efeitos colaterais da TARV e quantidade elevada de medicamentos) contribuem para a autogestão ineficaz da saúde em pessoas vivendo com HIV por causa da alta possibilidade de alterações gastrointestinais durante o período adaptativo fisiológico;
3. Fatores relacionados ao tempo de diagnóstico (inferior a dois anos) e de tratamento (inferior a um ano), negação da doença e *si-mismo* contribuem para a autogestão ineficaz da saúde em pessoas vivendo com HIV em razão da inexperiência em gerenciar uma condição crónica aliada ao estigma social no modo adaptativo de autoconceito;
4. Fatores resultantes de escolhas de vida (consumo de álcool e drogas ilícitas) contribuem para a autogestão ineficaz da saúde em pessoas vivendo com HIV devido à minimização da capacidade individual de cuidar de si causada pelo efeito de substância química no modo autoconceito;
5. Fatores de desempenho como a função social (baixa renda, atividade ocupacional e ambiente insalubre) e percepção social (estigma e medo de exposição do diagnóstico) contribuem para a autogestão ineficaz da saúde em pessoas vivendo com HIV por causa do processo de marginalização social no modo desempenho;
6. Fatores de interdependência consequentes das interações sociais (ausência de rede de apoio) ou do sistema de gestão (barreiras de acesso aos serviços de saúde, políticas públicas deficientes ou ausentes, baixo nível de escolaridade, indisponibilidade de medicamentos e qualidade da assistência prestada) contribuem para a autogestão ineficaz da saúde em pessoas vivendo com HIV em decorrência da exclusão social e sistêmica de populações vulneráveis;
7. A autogestão ineficaz da saúde em pessoas vivendo com HIV da saúde pode provocar danos sistêmicos (distúrbios neurológicos e/ou motores) e surgimento de doenças oportunistas;
8. Situações de agravo por autogestão ineficaz da saúde em pessoas vivendo com HIV aumentam a taxa de transmissibilidade do vírus, provocam resistência medicamentosa e elevam o número de recidivas de internação com probabilidade de prolongamento do período de estadia e aumento da morbimortalidade.

O Quadro 3 apresenta os fatores predisponentes, incapacitantes, precipitantes e de reforço originados dos antecedentes do DE “autogestão ineficaz da saúde” em pessoas vivendo com o HIV. Os 30 antecedentes foram agrupados da seguinte forma: 15 fatores predisponentes; 10 fatores incapacitantes; dois fatores precipitantes; e três fatores de reforço.

**Quadro 3:** Fatores predisponentes, incapacitantes, precipitantes e de reforço do diagnóstico de enfermagem “autogestão ineficaz da saúde” em pessoas vivendo com o HIV

Fator predisponente	
01	Uso de álcool
02	Uso de drogas ilícitas
03	Estigma e isolamento social
04	Baixo nível de escolaridade
05	Transtornos mentais
06	Baixa renda
07	Barreiras de acesso aos serviços de saúde
08	Ausência de rede de apoio
09	Raça (preta ou parda)
10	Negação da doença
11	Idade < 40 anos
12	Tempo de diagnóstico < 2 anos
13	Ambiente insalubre
14	Sexo masculino
15	Religião/crença contrária ao plano terapêutico
Fator incapacitante	
01	Efeitos colaterais da TARV
02	Esquecimento
03	Medo de exposição do diagnóstico
04	Quantidade elevada de medicamentos
05	Qualidade do cuidado e/ou relação com os profissionais de saúde
06	*Si-mismo*
07	Insegurança alimentar
08	Estágio clínico do HIV (OMS)
09	Coinfecção (hepatite e tuberculose)
10	Indisponibilidade de medicamentos
Fator precipitante	
01	Situações desestabilizadoras
02	Troca de regime terapêutico
Fator de reforço	
01	Atividade ocupacional
02	Tempo de tratamento < 1 ano
03	Ausência/deficiência de políticas públicas

## DISCUSSÃO

O conceito “Dificuldade, falha, abandono ou recusa em alcançar as metas pactuadas devido ao processo multifatorial e dinâmico que determina a ‘autogestão ineficaz da saúde’” construído com base nos atributos essenciais identificados na RE tem as características fundamentais que definem a autogestão ineficaz da saúde em pessoas vivendo com HIV. Essas características são indispensáveis para considerar a presença do diagnóstico de enfermagem nessa população, destacando suas propriedades básicas (comum a todas as instâncias) e distintivas (invariável) necessárias à formulação teórica subsequente.

A identificação dos atributos essenciais foram fundamentais para a formulação da Teoria de Médio Alcance (TMA) para a AIS-PVHIV por fornecer uma base sólida e científica para compreender os fatores que influenciam o processo de adaptação às medidas terapêuticas inerentes à doença crônica. Segundo Roy^(18)^ a capacidade humana de resposta continua aos estímulos internos e externos permitem à enfermagem prover auxílio no processo de adaptação.

Implementar o conceito de “autogestão ineficaz da saúde” na prática, alinhado com os atributos essenciais e com a teoria da adaptação, de Callista Roy^(18)^, envolve uma abordagem holística que inclui: avaliação individualizada das necessidades dos pacientes^(19)^, fornecimento de educação em saúde^(27)^, desenvolvimento de planos de cuidado personalizados^(19)^, estabelecimento de suporte contínuo^(33, 49)^, disponibilização de recursos e ferramentas^(47)^ e envolvimento familiar no processo de cuidado. Podemos observar que a abordagem holística produz resultados significativos no autogerenciamento em saúde entre PVHIV^(20, 23, 27)^.

Para além do conceito, a identificação de padrões e causas da AIS-PVHIV permitem compreender as variações no contexto clínico e social dos pacientes, o que possibilita ao Enfermeiro personalizar o cuidado a esta população específica, característica principal das TMAs. E assim, foi possível avaliar tais padrões e causas através do mapeamento dos antecedentes do DE pela RE, e em seguida, agrupá-los por modo adaptativo: fisiológico, de autoconceito, de função de papel e de interdependência^(18)^.

Os modos adaptativos constantes na teoria da adaptação^(18)^ representam as diferentes maneiras pelas quais um indivíduo responde e se adapta aos estímulos do ambiente. O modo fisiológico trata das necessidades físicas básicas. O modo de autoconceito refere-se à percepção e sentimentos que a pessoa tem sobre si mesma. O modo de função de papel abrange os papéis sociais que o indivíduo desempenha na vida, e o modo de interdependência envolve as relações sociais e o apoio que a pessoa recebe e oferece.

Agrupados no modo fisiológico, estão os antecedentes: raça preta ou parda; idade inferior a 40 anos; sexo masculino; presença de transtornos mentais; efeitos colaterais da terapia antirretroviral (TARV); elevada quantidade de medicamentos; apresentação farmacológica dos medicamentos e esquecimento quanto ao uso da TARV.

A idade inferior a 40 anos foi associada ao DE “autogestão ineficaz da saúde” em pessoas vivendo com HIV devido a uma considerável menor maturidade e experiência para lidar com decisões em saúde pertinentes às condições crônicas, como citado por alguns autores^(20, 28, 36, 39, 42, 46, 48)^. O sexo masculino foi frequentemente apontado entre os autores^(20, 21, 26, 32, 39)^ como fator predisponente, havendo várias razões para a menor adesão à TARV por pessoas desse sexo^(26)^. O número limitado de serviços direcionados à saúde do homem e fatores psicossociais como masculinidade, cultura e estigma contribuem para a perda de seguimento/acompanhamento de tal população.

A presença de transtornos mentais também foi apontada como fator predisponente, sendo a depressão^(20, 22, 26, 31, 36, 37, 38, 41, 43, 47)^ e a ansiedade^(28, 31, 32, 38, 42, 47)^ as mais referenciadas. Um estudo transversal canadense^(33)^ mostrou relação positiva entre a depressão e ansiedade e o diagnóstico de HIV/aids, com incidência duas a três vezes maior que no restante da população. Outra pesquisa explica que os diagnósticos de depressão e ansiedade podem interferir na autogestão ineficaz da saúde em pessoas vivendo com HIV pelo grau de comprometimento desses transtornos na capacidade de cuidar de si, incluindo seguir tratamentos complexos^(22, 31)^.

Além disso, os fatores associados ao medicamento (efeitos colaterais da TARV, elevada quantidade de medicamentos e a apresentação farmacológica) podem desestimular a adesão e/ou continuidade terapêutica^(21, 28, 29, 33, 37, 38, 47, 48, 49)^. Os efeitos colaterais da TARV frequentemente correlatados envolvem distúrbios metabólicos, neurológicos e gastrointestinais (náuseas, diarreia, dor abdominal e vômitos)^(29, 37, 38)^, sendo o último fortemente afetado pela apresentação dos medicamentos (odor, tamanho e sabor)^(38)^ e quantidade acentuada de medicamentos relacionada ao medo de interação medicamentosa entre os fármacos^(33, 49)^, essencialmente na presença de comorbidades^(28)^. Por sua vez, o esquecimento foi associado a sobrecarga diária (trabalho, estudo, família) do paciente^(21, 29)^, não utilização de mecanismos de reforço à dosagem^(35, 44, 48)^, e ausência de rede de apoio motivador^(38)^.

No tocante ao modo autoconceito, foram identificados os seguintes fatores: tempo de diagnóstico inferior a dois anos; tempo de tratamento inferior a um ano; uso de álcool; uso de substâncias ilícitas; negação da doença; expectativa de morte iminente; tomada de decisões contrárias ao plano terapêutico; falsa sensação de melhora clínica; sucessivos resultados laboratoriais positivos; religião ou crença contrárias ao tratamento; e uso secreto das medicações.

A maior propensão ao DE AIS-PVHIV em pessoas com tempo de diagnóstico inferior a dois anos e tempo de tratamento inferior a um ano podem ser justificada por quatro diferentes fatores: pelo impacto emocional de um diagnóstico recente^(37, 38, 39, 40)^; pela falta de experiência em gerenciar uma condição crônica^(48, 49)^; pelo estigma social que dificulta o acesso ao suporte necessário^(19, 35, 48, 49)^; e, pela complexidade dos esquemas de medicação e seus efeitos colaterais iniciais também^(37, 40)^ podem ser desafiadores para pacientes recém-diagnosticados.

Neste ponto, ressaltamos os antecedentes “uso de álcool” e “abuso de drogas ilícitas” visto por todos os autores^(22, 24, 26, 29, 38, 39, 42, 43, 44, 48, 49)^, que os mencionaram como mecanismo mais incidente de enfrentamento diante da descoberta do diagnóstico de HIV, com propensões significativas ao DE “autogestão ineficaz da saúde” em pessoas vivendo com HIV. De acordo com autores^(26, 38)^, esses abusos podem ser um meio de “fuga” aos transtornos psicológicos anteriormente discutidos, que, por sua vez, minimizam a capacidade individual de cuidar de si, ainda que sob uso ocasional. Além disso, há evidências^(44)^ que correlacionam o abuso de álcool entre PVHIV à fase inicial após o diagnóstico de HIV, quando se dá a negação da doença^(21, 25, 41)^ e o *si-mismo* ^(21, 48)^ com maior frequência, ambos também identificados como antecedentes do DE.

A religião ou crença contrárias ao tratamento podem oferecem modos alternativos de “cura” e também se relacionam a negligência^(21, 35, 44)^. a negligência da necessidade contínua de adesão ao tratamento, baseando-se apenas em sintomas temporários ou melhorias aparentes, que levam à complacência e à interrupção do tratamento^(35)^.

No modo de interdependência, foram identificados os seguintes antecedentes: barreiras no acesso aos serviços de saúde; falta ou inadequação de políticas públicas; baixo nível de escolaridade; indisponibilidade de medicamentos; falhas diagnósticas durante o pré-natal; supervisão ineficiente do tutor; troca do regime terapêutico; ausência de uma rede de apoio; qualidade do cuidado e relacionamento com os profissionais de saúde; dificuldade de agendar consultas e procedimentos; e isolamento social.

A presença de barreiras de acesso aos serviços de saúde pode estar relacionada a dificuldades na obtenção de cuidados periódicos, monitoramento da carga viral e tratamento oportuno devido a fatores socioeconómicos, geográficos, culturais, de deslocamento e ausência ou deficiência de políticas públicas que garantam o acesso à clientela^(19, 22, 23, 25, 26, 27, 32, 36, 38, 40, 42, 43, 44, 45, 46, 49)^. Tais barreiras incluem outros antecedentes apontados no estudo em tela como: indisponibilidade de medicamentos^(24, 27, 30, 41)^ e trocas sucessivas de regime terapêutico pelas redes de distribuição^(33, 39)^, dificuldade para agendamento de consultas e procedimentos^(43, 48)^, e a qualidade do cuidado e relação com os profissionais de saúde^(25, 26, 27)^. Todos esses fatores foram apontados como risco para pausas e descontinuidades de tratamento com a TARV^(23)^, automedicação inadequada^(25)^, redução da eficácia do tratamento e aumento do risco de desenvolvimento de resistência viral^(44, 49)^.

O antecedente “baixo nível de escolaridade” está relacionado ao DE AIS-PVHIV por limitar a compreensão do indivíduo sobre a doença, importância do tratamento e os cuidados necessários para gerenciar a condição de forma eficaz^(20, 22, 24, 30, 31, 32, 36, 40, 42, 44, 49)^. A baixa escolaridade afeta o entendimento de instruções médicas, a capacidade de decisão sobre o próprio regime terapêutico e o acesso aos serviços de saúde devido a barreiras linguísticas e culturais^(36)^.

Entre os fatores que fortalecem a presença do antecedente “ausência de uma rede de apoio” e interferem na presença do DE “autogestão ineficaz da saúde” em pessoas vivendo com o HIV, os mais relatados foram: apoio emocional estimulante^(19, 21)^; auxílio de informações relevantes em saúde^(44)^; acompanhamento e monitoramento de consultas, exames e medicamentos^(25, 37, 40)^; ser uma fonte potencial de incentivo e motivação^(44)^; e ser fonte de acesso a recursos (financeiros e de suporte) necessários à continuidade da autogestão da saúde^(32)^.

Por fim, no modo desempenho de papel, foram identificados: baixa renda; atividade ocupacional; ambiente insalubre; estigma social e isolamento; e medo de exposição do diagnóstico. Baixa renda e ocupações precárias se coadunam ao DE por limitar o acesso a recursos de saúde, medicamentos e suporte psicológico profissional^(20, 30, 31, 32, 34, 36, 39, 40, 42, 43, 44, 48)^. Ambientes insalubres podem agravar problemas de saúde existentes e dificultar a manutenção de uma rotina de autocuidado como o caso de pessoas privadas de liberdade^(23, 43)^, transexuais^(28)^ e profissionais do sexo^(29)^. O estigma social foi frequentemente associado ao DE por induzir ao isolamento e à rejeição de ajuda^(19, 23, 25, 28, 29, 31, 32, 34, 35, 40, 41, 47)^, enquanto o medo de exposição do diagnóstico^(19, 29, 31, 34, 35, 41, 49)^ pode impedir a busca por cuidados necessários^(19, 34)^. Esses fatores interagem de maneira complexa potencializando um ambiente nocivo e criando barreiras substanciais à adaptação de PVHIV ao plano terapêutico estabelecido comuns ao processo de marginalização social.

No foco da prática de enfermagem para o modo fisiológico, as intervenções devem concentrar-se na supervisão periódica, considerando que esses fatores, embora passíveis de controle, são variados e independem diretamente das boas práticas em saúde, apesar de monitoráveis. Para o modo autoconceito, a definição de metas de forma dialógica estabelece um vínculo de confiança e integra a prática à realidade vivenciada pelo paciente, mitigando as dificuldades resultantes de um regime terapêutico muitas vezes inadequado, verticalizado e generalista. Quanto ao modo de desempenho de papel, é relevante um acompanhamento multidisciplinar e interdisciplinar fundamentado em uma abordagem integralista que considera a complexidade do processo saúde-doença e suas consequências. Por último, no modo de interdependência, é fundamental garantir o acesso universal, integral, equânime e humanizado aos serviços de saúde, para evitar que as desigualdades sociais e a prevalência do modelo flexneriano interfiram na autogestão em saúde das pessoas vivendo com HIV.

A identificação dos fatores consequentes é crucial para determinar a presença do DE AIS-PVHIV, pois esses fatores fornecem sinais e evidências objetivas de que a autogestão está comprometida^(8, 14)^. Eles ajudam a compreender os desafios específicos enfrentados pelos pacientes. Compreender esses fatores permite aos enfermeiros realizarem uma avaliação mais precisa e desenvolver intervenções personalizadas para melhorar a autogestão^(3, 4)^. Além disso, a identificação dos fatores consequentes pode orientar políticas de saúde e programas de suporte, promovendo uma abordagem mais holística e eficaz no cuidado dessas pessoas, garantindo melhor adesão ao tratamento e qualidade de vida^(18)^.

Por fim, o pictograma foi pensado com a finalidade de facilitar a retenção das informações extraídas do estudo em questão^(8)^. Em algumas situações, a comunicação entre pacientes, profissionais de saúde e familiares pode ser dificultada por diferenças culturais, etárias e educacionais. Nesse sentido, o material educacional sintetizado neste estudo foi elaborado para mitigar o risco de falhas de comunicação^(8)^ entre os atores envolvidos nesse processo, visando aprimorar tanto a compreensão dos profissionais de enfermagem quanto a adesão dos pacientes.

Com isso, o desenvolvimento da TMA em tela, o enfermeiro reconhecerá os princípios deste fenómeno, já que permeia uma variedade de fatores demográficos e psicossociais, onde ações tecnológicas do cuidado podem promover uma autogestão eficaz do tratamento^(^22-24^)^. O constructo desenvolvido deverá exercer um impacto positivo na prevenção de comorbidades e complicações às quais as pessoas vivendo com HIV estão expostas, resultando, assim, em uma melhoria na sua qualidade de vida.

### Limitações do estudo

Frequentemente, as teorias de médio alcance realizadas com base em revisões de escopo enfrentam algumas limitações notáveis. Uma das principais limitações é a heterogeneidade das fontes e dos dados incluídos, o que pode resultar em inconsistências e dificuldades na generalização dos achados. Além disso, a qualidade variada dos estudos utilizados na revisão pode impactar a robustez das conclusões, já que algumas pesquisas podem apresentar vieses metodológicos ou limitações próprias. Outro desafio é a complexidade na síntese de múltiplos tipos de evidências (quantitativas e qualitativas), podendo dificultar a construção de uma teoria coesa e abrangente.

Finalmente, a dependência de dados secundários pode limitar a profundidade da análise, pois os dados originais não são reavaliados diretamente, podendo deixar lacunas importantes na compreensão completa do fenómeno estudado. Essas limitações ressaltam a necessidade de abordagens críticas e rigorosas na condução de revisões de escopo para o desenvolvimento de teorias de médio alcance.

A limitação deste estudo relaciona-se à utilização exclusiva de artigos de acesso aberto na revisão de escopo, o que pode ter resultado no pequeno tamanho da amostra. Outra limitação foi a ausência das etapas de validação necessárias para a verificação dos pressupostos teóricos contra dados empíricos, permitindo ajustes e refinamentos necessários.

### Contribuições para área de Enfermagem

A TMA desenvolvida apresenta uma contribuição importante para o cuidado das PVHIV, uma vez que favorece o julgamento clínico e raciocínio diagnóstico dos enfermeiros. Consequentemente, ela contribui para o planejamento de ações de promoção à saúde a essa população e para a prevenção de agravos relacionados à autogestão ineficaz da saúde. Ressalta-se que esse fenómeno pode estar associado à falta de adesão ao uso de antirretrovirais, contribuindo para o aumento de novos casos da doença, internações por complicações e mortalidade relacionada a essa enfermidade. Além disso, avança no conhecimento da ciência da enfermagem, por permitir o estabelecimento de um aporte teórico sobre o fenómeno estudado, mostrando-se necessária como evidência para a atualização da taxonomia da NANDA-I. Por fim, os resultados podem servir de base em estudos posteriores, a fim de avançar com a validação de conteúdo e clínica.

## CONCLUSÕES

O desenvolvimento da teoria de médio alcance permitiu maior compreensão das relações de causalidade entre os conceitos que abrangem o diagnóstico de enfermagem “autogestão ineficaz da saúde” em pessoas vivendo com HIV. Identificaram-se cinco atributos essenciais, 35 antecedentes e 19 consequentes clínicos do diagnóstico de enfermagem estudado. Para facilitar a compreensão desses indicadores, foi criado um pictograma ilustrado que abrange a relação dos conceitos (atributos, antecedentes e consequentes) entre si e destes com a teoria da adaptação, de Callista Roy. Por fim, foram desenvolvidas sete proposições.

O constructo desenvolvido deverá impactar positivamente na prevenção de comorbidades e agravos à que estão sujeitas às pessoas vivendo com HIV e consequentemente em sua qualidade de vida. Sob uma perspectiva mais abrangente, impactará diretamente na redução dos custos associados às internações recorrentes e aos procedimentos de alto custo necessários para o tratamento de infecções e condições oportunistas às quais indivíduos com o diagnóstico de enfermagem Autogestão Ineficaz da Saúde estão predispostos.

Portanto, conclui-se que a teoria de médio alcance concebida para o diagnóstico de enfermagem “autogestão ineficaz da saúde” em pessoas vivendo com HIV oportunizará contribuições relevantes para a promoção da saúde e qualidade de vida dessa população específica.

## Data Availability

Não se aplica.
